# jMorp updates in 2020: large enhancement of multi-omics data resources on the general Japanese population

**DOI:** 10.1093/nar/gkaa1034

**Published:** 2020-11-12

**Authors:** Shu Tadaka, Eiji Hishinuma, Shohei Komaki, Ikuko N Motoike, Junko Kawashima, Daisuke Saigusa, Jin Inoue, Jun Takayama, Yasunobu Okamura, Yuichi Aoki, Matsuyuki Shirota, Akihito Otsuki, Fumiki Katsuoka, Atsushi Shimizu, Gen Tamiya, Seizo Koshiba, Makoto Sasaki, Masayuki Yamamoto, Kengo Kinoshita

**Affiliations:** Tohoku Medical Megabank Organization, Tohoku University, 2-1 Seiryo-machi, Aoba-ku, Sendai, Miyagi 980-8573, Japan; Tohoku Medical Megabank Organization, Tohoku University, 2-1 Seiryo-machi, Aoba-ku, Sendai, Miyagi 980-8573, Japan; Tohoku University Advanced Research Center for Innovations in Next-Generation Medicine, Tohoku University, 2-1 Seiryo-machi, Aoba-ku, Sendai, Miyagi 980-8573, Japan; Iwate Tohoku Medical Megabank Organization, Disaster Reconstruction Center, Iwate Medical University, 1-1-1 Idaidori, Yahaba-cho, Shiwa-gun, Iwate 028-3694, Japan; Tohoku Medical Megabank Organization, Tohoku University, 2-1 Seiryo-machi, Aoba-ku, Sendai, Miyagi 980-8573, Japan; Graduate School of Information Sciences, Tohoku University, 6-3-09 Aramaki aza Aoba, Aoba-ku, Sendai, Miyagi 980-8579, Japan; Tohoku Medical Megabank Organization, Tohoku University, 2-1 Seiryo-machi, Aoba-ku, Sendai, Miyagi 980-8573, Japan; Tohoku Medical Megabank Organization, Tohoku University, 2-1 Seiryo-machi, Aoba-ku, Sendai, Miyagi 980-8573, Japan; Graduate School of Medicine, Tohoku University, 2-1 Seiryo-machi, Aoba-ku, Sendai, Miyagi 980-8575, Japan; Tohoku Medical Megabank Organization, Tohoku University, 2-1 Seiryo-machi, Aoba-ku, Sendai, Miyagi 980-8573, Japan; Tohoku Medical Megabank Organization, Tohoku University, 2-1 Seiryo-machi, Aoba-ku, Sendai, Miyagi 980-8573, Japan; Tohoku University Advanced Research Center for Innovations in Next-Generation Medicine, Tohoku University, 2-1 Seiryo-machi, Aoba-ku, Sendai, Miyagi 980-8573, Japan; Tohoku Medical Megabank Organization, Tohoku University, 2-1 Seiryo-machi, Aoba-ku, Sendai, Miyagi 980-8573, Japan; Tohoku University Advanced Research Center for Innovations in Next-Generation Medicine, Tohoku University, 2-1 Seiryo-machi, Aoba-ku, Sendai, Miyagi 980-8573, Japan; Tohoku Medical Megabank Organization, Tohoku University, 2-1 Seiryo-machi, Aoba-ku, Sendai, Miyagi 980-8573, Japan; Graduate School of Information Sciences, Tohoku University, 6-3-09 Aramaki aza Aoba, Aoba-ku, Sendai, Miyagi 980-8579, Japan; Tohoku Medical Megabank Organization, Tohoku University, 2-1 Seiryo-machi, Aoba-ku, Sendai, Miyagi 980-8573, Japan; Tohoku University Advanced Research Center for Innovations in Next-Generation Medicine, Tohoku University, 2-1 Seiryo-machi, Aoba-ku, Sendai, Miyagi 980-8573, Japan; Graduate School of Medicine, Tohoku University, 2-1 Seiryo-machi, Aoba-ku, Sendai, Miyagi 980-8575, Japan; Graduate School of Information Sciences, Tohoku University, 6-3-09 Aramaki aza Aoba, Aoba-ku, Sendai, Miyagi 980-8579, Japan; Tohoku Medical Megabank Organization, Tohoku University, 2-1 Seiryo-machi, Aoba-ku, Sendai, Miyagi 980-8573, Japan; Graduate School of Medicine, Tohoku University, 2-1 Seiryo-machi, Aoba-ku, Sendai, Miyagi 980-8575, Japan; Tohoku Medical Megabank Organization, Tohoku University, 2-1 Seiryo-machi, Aoba-ku, Sendai, Miyagi 980-8573, Japan; Graduate School of Medicine, Tohoku University, 2-1 Seiryo-machi, Aoba-ku, Sendai, Miyagi 980-8575, Japan; Iwate Tohoku Medical Megabank Organization, Disaster Reconstruction Center, Iwate Medical University, 1-1-1 Idaidori, Yahaba-cho, Shiwa-gun, Iwate 028-3694, Japan; Institute for Biomedical Sciences, Iwate Medical University, 1-1-1 Idaidori, Yahaba-cho, Shiwa-gun, Iwate 028-3694, Japan; Tohoku Medical Megabank Organization, Tohoku University, 2-1 Seiryo-machi, Aoba-ku, Sendai, Miyagi 980-8573, Japan; Graduate School of Medicine, Tohoku University, 2-1 Seiryo-machi, Aoba-ku, Sendai, Miyagi 980-8575, Japan; Tohoku Medical Megabank Organization, Tohoku University, 2-1 Seiryo-machi, Aoba-ku, Sendai, Miyagi 980-8573, Japan; Tohoku University Advanced Research Center for Innovations in Next-Generation Medicine, Tohoku University, 2-1 Seiryo-machi, Aoba-ku, Sendai, Miyagi 980-8573, Japan; Iwate Tohoku Medical Megabank Organization, Disaster Reconstruction Center, Iwate Medical University, 1-1-1 Idaidori, Yahaba-cho, Shiwa-gun, Iwate 028-3694, Japan; Institute for Biomedical Sciences, Iwate Medical University, 1-1-1 Idaidori, Yahaba-cho, Shiwa-gun, Iwate 028-3694, Japan; Tohoku Medical Megabank Organization, Tohoku University, 2-1 Seiryo-machi, Aoba-ku, Sendai, Miyagi 980-8573, Japan; Tohoku University Advanced Research Center for Innovations in Next-Generation Medicine, Tohoku University, 2-1 Seiryo-machi, Aoba-ku, Sendai, Miyagi 980-8573, Japan; Graduate School of Medicine, Tohoku University, 2-1 Seiryo-machi, Aoba-ku, Sendai, Miyagi 980-8575, Japan; Tohoku Medical Megabank Organization, Tohoku University, 2-1 Seiryo-machi, Aoba-ku, Sendai, Miyagi 980-8573, Japan; Tohoku University Advanced Research Center for Innovations in Next-Generation Medicine, Tohoku University, 2-1 Seiryo-machi, Aoba-ku, Sendai, Miyagi 980-8573, Japan; Graduate School of Information Sciences, Tohoku University, 6-3-09 Aramaki aza Aoba, Aoba-ku, Sendai, Miyagi 980-8579, Japan

## Abstract

In the Tohoku Medical Megabank project, genome and omics analyses of participants in two cohort studies were performed. A part of the data is available at the Japanese Multi Omics Reference Panel (jMorp; https://jmorp.megabank.tohoku.ac.jp) as a web-based database, as reported in our previous manuscript published in *Nucleic Acid Research* in 2018. At that time, jMorp mainly consisted of metabolome data; however, now genome, methylome, and transcriptome data have been integrated in addition to the enhancement of the number of samples for the metabolome data. For genomic data, jMorp provides a Japanese reference sequence obtained using *de novo* assembly of sequences from three Japanese individuals and allele frequencies obtained using whole-genome sequencing of 8,380 Japanese individuals. In addition, the omics data include methylome and transcriptome data from ∼300 samples and distribution of concentrations of more than 755 metabolites obtained using high-throughput nuclear magnetic resonance and high-sensitivity mass spectrometry. In summary, jMorp now provides four different kinds of omics data (genome, methylome, transcriptome, and metabolome), with a user-friendly web interface. This will be a useful scientific data resource on the general population for the discovery of disease biomarkers and personalized disease prevention and early diagnosis.

## INTRODUCTION

To realize personalized healthcare and personalized medicine for a specific population, an important step is to collect multi-omics-level information of the general population for each ethnic group, because it is widely known that diversity exists within the genetic and molecular status of an individual, which affects their response to medication and side-effects of various drugs. To employ genetic and molecular information for personalized healthcare, constructing population-specific reference panels is a fundamental step, using which a group of data can be compared and a standard of molecular and genetic status in the population can be obtained. To address this, several projects such as the UK Biobank (1) in the United Kingdom, deCODE (2) in Iceland, and LifeLines-deep study (3) have been conducting large-scale cohort-based multi-omics studies including large-scale genome analyses. In the UK Biobank cohort, a total of 500,000 genotyping experiments with single nucleotide polymorphism (SNP) arrays were performed by 2018 (4), and now, whole-genome analyses of all participants of the UK Biobank are being conducted by deCODE genetics Inc. in Iceland and the Welcome Sanger Institute in the United Kingdom. In addition, the world's largest whole-genome repository is expected to be developed by summer in 2021. The LifeLines-deep is a sub-project of the LifeLines cohort study with a focus on 165,000 participants with 30 years follow-up and multi-omics analyses of 1,500 participants, which aims to obtain whole-genome genotype, whole-genome methylation, whole-genome gene expression, plasma and exhaled breath metabolome, and gut microbiome data (5–7).

For the Japanese population, The Tohoku Medical Megabank Organization (ToMMo) and Iwate Medical Megabank Organization (IMM), which were established just after the Great East Japan Earthquake on 11 March 2011, initiated two prospective cohort studies to realize constructive regeneration from the disaster and to deliver advanced medication to people affected by the tsunami of the earthquake, as a national project called The Tohoku Medical Megabank (TMM) project (8). One cohort is a population‐based adult cohort (9), and the other is a birth and three‐generation cohort (10). For the TMM project, approximately 150,000 participants in total were successfully recruited for the two cohort studies. Using the two cohorts, we constructed an integrated biobank by collecting biological specimens and performed typical genome and omics analyses by using the samples in the biobank (11,12); then, we distributed the data upon requests after the approval of the ethical committee and the data access committee under strict security control to protect the privacy of the participants (13). In addition, to distribute various kinds of data as open data for the scientific community, which can be used as reference data for the Japanese population, we constructed a web-based database of data obtained in the TMM project called jMorp: Japanese Multi-Omics Reference Panel. jMorp was first launched in July 2015 as a database that provides integrated analysis results of metabolome and proteome data from approximately 500 plasma samples (14). In the initial version of jMorp, we focused on metabolome data obtained by proton NMR and LC–MS and proteome data with nano LC–MS; we released distributions of concentrations of 37 metabolites measured by NMR and distributions of peak intensities of 257 characterized metabolites by LC–MS and observed frequencies of 256 abundant proteins by LC–MS.

Since the first release of jMorp, we have rapidly increased the amount of data and added different kinds of information in addition to proteome and metabolome data. In the current updated version, we mainly enhanced the following four features compared with the previous report: (i) a reference genome specially constructed for the Japanese population has been added, (ii) genome variation data determined by whole-genome sequencing of >8,300 healthy volunteers have been integrated, (iii) transcriptome and methylome data have been integrated and (iv) the amount of metabolome data has been increased to 755 metabolites from measured plasma samples taken from >23,000 healthy volunteers. Thus, the jMorp database has now become a reference panel for genomics, proteomics, and metabolomics. In other words, it provides a comprehensive collection of information on human life sciences for the Japanese population. Almost all jMorp data are available from user-friendly web interfaces, and it allows researchers to easily check the diversity in the Japanese population across multiple levels of genome-omics information. As far as we know, this is the first integrated database including multiple omics layers in a single population and will be a good model database for other populations. In this paper, we focus on the updates of the jMorp database in 2020.

## OVERVIEW OF JMORP: AVAILABLE DATA AND FUNCTIONALITIES

### Summary of datasets available from jMorp

**Figure 1.**
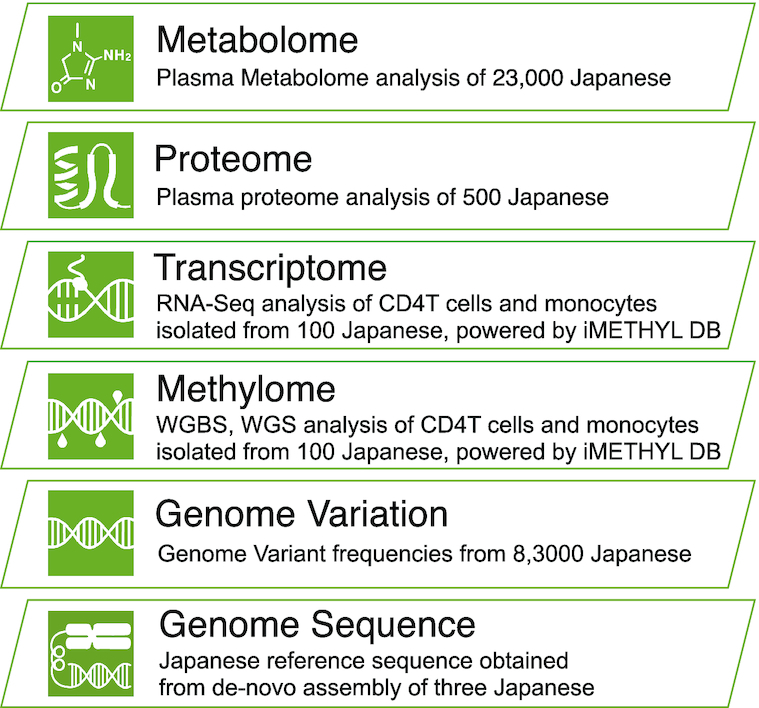
Overview of the main datasets included in jMorp. Schematic representation of the dataset in jMorp, overlaid on top of a hierarchical diagram that mimics the central dogma.

Figure 1 shows an overview of the main datasets included in jMorp, and the number of samples per dataset is provided in Supplementary Table S1(a). As of 2015, jMorp included metabolome and proteome datasets from analysis results of 501 Japanese individuals; however, the current release covers multiple layers beyond the metabolome, such as the genome. jMorp also provides information other than analyzed data, as shown in Supplementary Table S1(b), which includes indispensable data resources for genomic analyses for the Japanese population. As shown in Supplementary Table S1, jMorp contains a wide variety of data, which enables us to obtain data on multiple layers from the genome, which is the starting point of the central dogma, to the metabolome, which is adjacent to the phenotype layer. The top page of jMorp has a layer diagram clarifying our concept of multi-layered data according to the central dogma, and users can access the data by clicking on each layer. In addition, most of the data provided by jMorp is available for bulk download from the download page for further secondary analyses by users. Most of the data released from jMorp can be freely downloaded. However, some data, such as genotype frequency information calculated from whole genome analysis, requires agreement to the Data Transfer Agreement (DTA) for security reasons, such as how it is handled after download. The jMorp website has implemented a login mechanism using ORCID, and uses it to perform per-user data access control. As for the data that requires DTA, it is also available for free download. The following sections provide details of each dataset and its usage.

### Japanese reference genome JG2

JG2 is the second version of the Japanese population-specific reference genome (15). The reference genome, such as GRCh38, has been an invaluable resource for human genetics and is maintained by the Genome Reference Consortium; however, some population-specific sequences may not be represented by a single reference genome. To overcome this limitation, we constructed a reference genome for the Japanese population-called JG1 by integrating three *de novo* human genome assemblies based on long- and short-read and next-generation optical mapping technologies. JG2 is an update of the Japanese reference genome and is constructed from the same three individuals; however, it is reconstructed using new sequencing data such as nanopore long-read and Hi-C technologies and a new computational assembly method. The details of the construction methods are described in the technical note available from the Downloads section at the jMorp website. The sequence FASTA file, along with chain files, against the other reference genomes can be downloaded.

**Figure 2.**
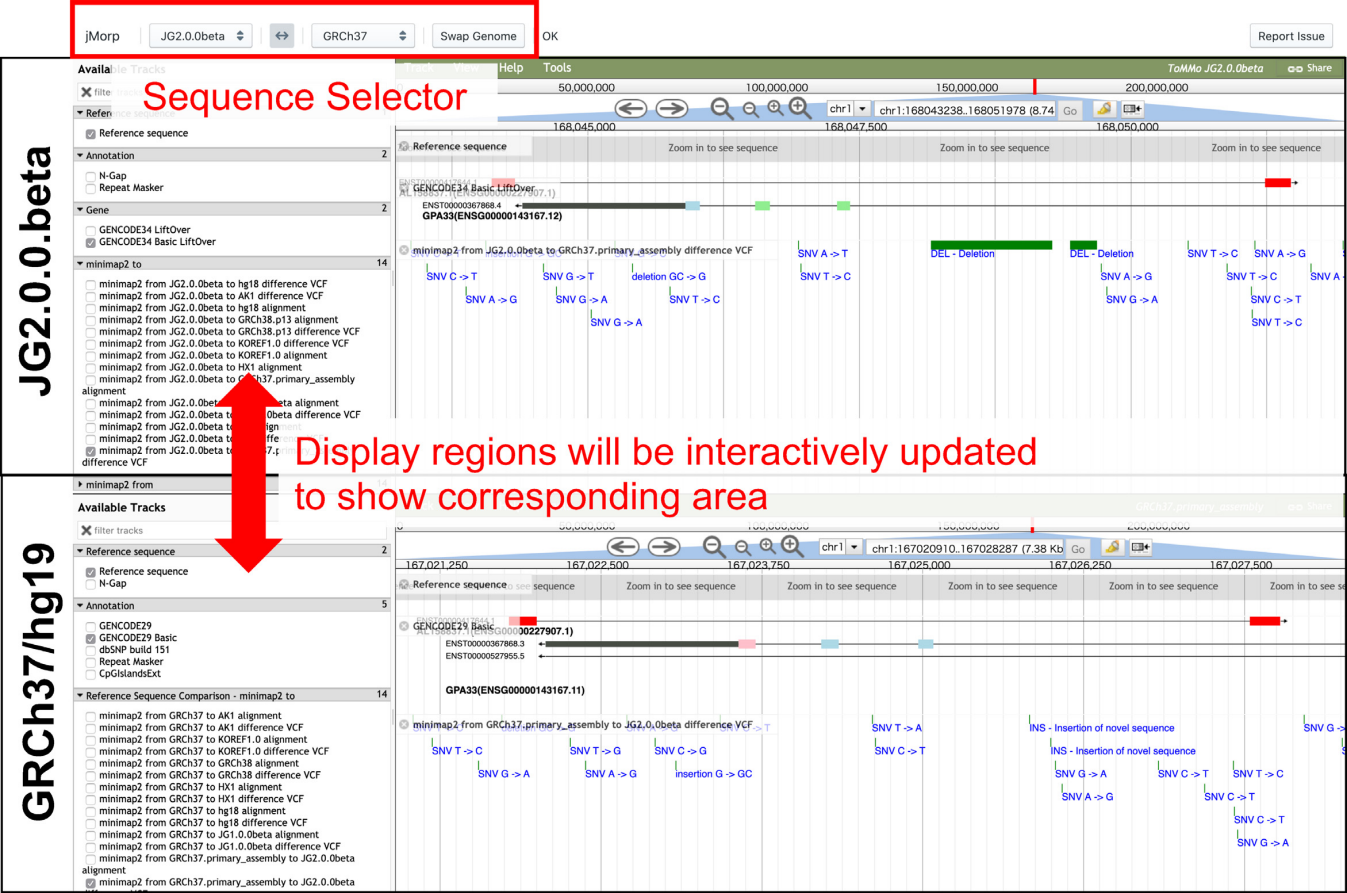
JBrowse-based browser for comparison with JG2 and other genomes. In the figure, a genome browser with the JG2 sequence in the upper row and a genome browser with the GRCh37 sequence in the lower row are shown. The display areas of each genome browser can be changed by dragging the mouse on the genome browser. When the display region of one genome browser is changed, the display region of the other genome browser is automatically updated in conjunction with the other genome browser. Using the select box in the top row of the screen, the reference genome sequences shown in the two genome browsers can be changed. In the top half view of Figure 2, there is a track named ‘minimap2 from JG2.0.0.beta to GRCh37.primary_assembly difference VCF’, where alignment of GRCh37 sequence onto JG2 sequence is shown. In Figure 2, two dark green bars are shown, indicating that there are two deletions in GRCh37 compared with the JG2.

In the jMorp genome sequence layer, a JBrowse (16)-based genome browser allows for browsing of JG2 and comparison with other reference genomes such as GRCh37 and GRCh38 (Figure 2). Comparisons between the reference genomes were made possible by having multiple JBrowses arranged in a single window and linked to view the corresponding regions simultaneously. Available annotation tracks include genic regions based on lifted-over GENCODE annotations, aligned genomic regions as well as differences within those regions against other reference genomes, repetitive regions defined by RepeatMasker software, and N-gap regions.

### 8.3KJPN whole-genome variation panel

The TMM project has finished sequencing nearly 9,000 individuals from a Japanese population. In the genome variation layer, we provide an allele frequency panel of 8,380 individuals which has been obtained after removing related samples from the analyzed whole-genome data, which we call the 8.3KJPN whole-genome variation panel. As shown in Supplementary Table S2, most of the genome data in 8.3KJPN are mainly from the samples collected from participants of the TMM cohorts in Miyagi and Iwate prefectures, and genome data from western Japan were also included in addition to those from participants residing in the Tohoku region as a collaboration with several external cohorts. Details of the genome analyses and regional genetic differences among Japanese populations have already been described by Tadaka *et al.* (17) and Yasuda *et al.* (11), respectively.

**Figure 3.**
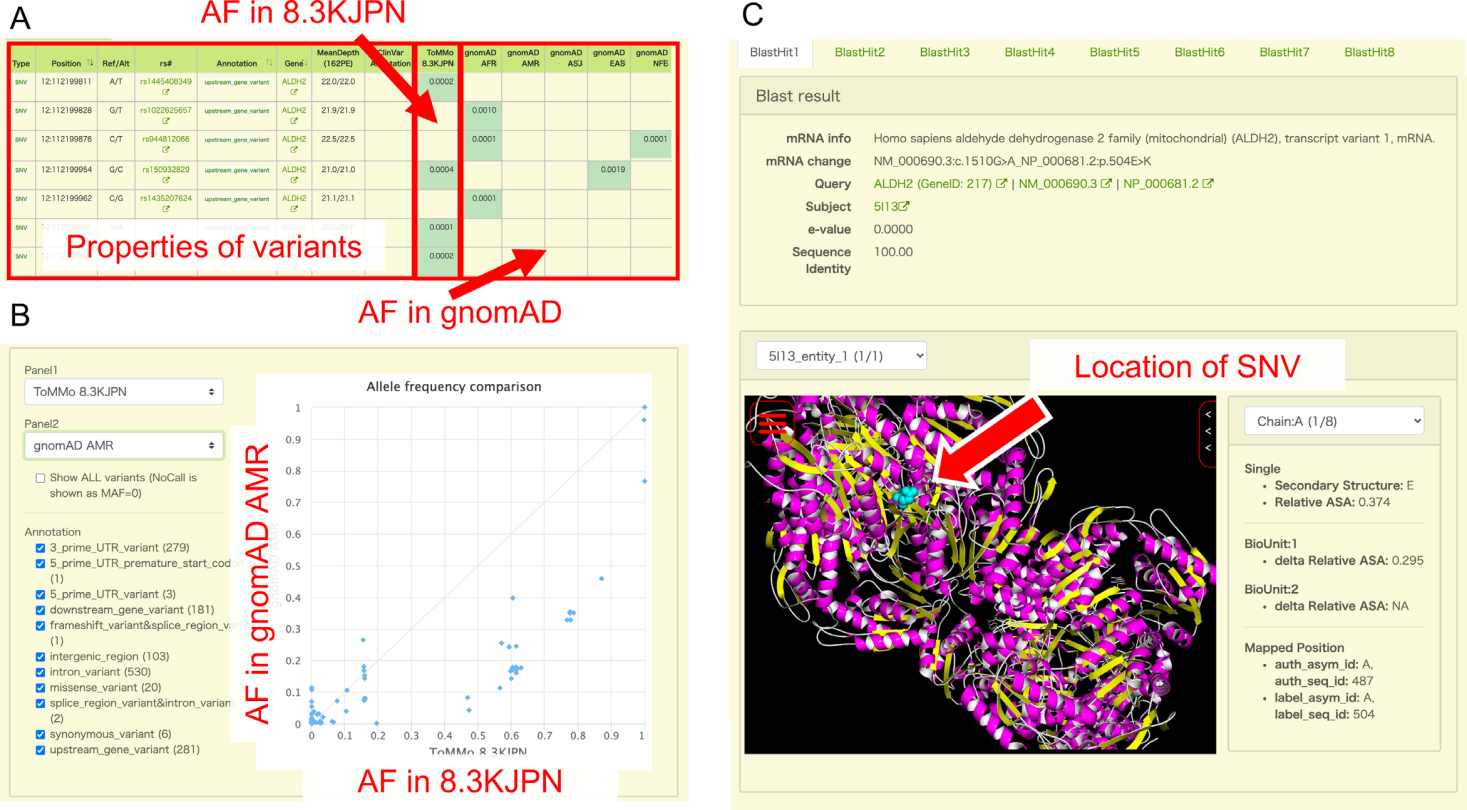
(**A**) Example of a search result table when a user searches for genomic variants on the ALDH2 gene. The basic properties of the variants are displayed on the left side of the table, and the allele frequency (AF) in 8.3KJPN and gnomAD populations are displayed on the right side. (**B**) Comparison of 8.3KJPN and gnomAD AMS (Admixed American) using genomics variants on ALDH2 gene as an example by the AF comparison tool. In the scatter plot on the right of the figure, one dot corresponds to one SNV, and the horizontal and vertical axes represent the frequency of each SNV at 8.3KJPN and gnomAD AMR, respectively. Users can freely change populations displayed as horizontal and vertical axes using the selector at the top left. The SNVs displayed on the scatter plot can be filtered using functional annotation of each SNV. (**C**) The Structure Mapping page displays the available protein structure data for a particular SNV. If multiple isoforms of RefSeq are mapped on an SNV, we choose a representative isoform by alphabetically sorting their descriptions and selecting the first isoform, e.g. if the isoforms of a gene are numbered, ‘transcript variant 1’ is selected. ‘BlastHit’ tabs can be used to switch the structures of different protein sequences, and they are sorted by descending BLAST scores. Identical protein sequences in the whole PDB are grouped into a single BlastHit tab, and each entity (unique sequence in a PDB entry) in the group can be selected using the ‘PDB entity’ dropdown list. The ‘Chain’ dropdown list can be used to highlight the substituted amino acid of each chain. Below the ‘Chain’ dropdown list, the secondary structure and ASA of the residue is shown. The ASA of each residue is normalized by the ASA of the same residue type ‘X’ in an extended Gly-X-Gly conformation (Relative ASA). ‘delta Relative ASA’ is the difference in Relative ASA of a residue between in the state where each protein chain is isolated and in the state of biological unit. The MolMil viewer can be used to interactively indicate the positions of the substituted amino acid in the 3D structure.

By using the web interface provided by jMorp, users can obtain a table of variant information by entering several search criteria such as gene name, rsID (dbSNP ID), or chromosomal position. The search result table (Figure 3A) contains allele frequencies in 8.3KJPN with rsIDs, functional and disease annotations, average depth of whole-genome analysis, and allele frequencies in several other populations in the Genome Aggregation Database (gnomAD) (18). In addition, comparisons of allele frequencies with other populations in a scatter plot can also be obtained by pushing a ‘plot button’ above the search result table, which will easily give users an overview of ethnic differences in allele frequencies. Figure 3B shows the comparison of 8.3KJPN and gnomAD AMS (Admixed American) using genomics variants on the ALDH2 gene as an example. In the plot, a point corresponds to an SNV or indel, and the horizontal and vertical axes show the allele frequencies for 8.3KJPN and gnomAD AMS, respectively. If a point on the plot lies on a diagonal line, it means that the frequencies in the two selected populations are equal, whereas a variant that lies off the diagonal line means that the difference in frequencies between the two selected ethnic groups is large. jMorp also provides a tool to inspect the position of a variant on its corresponding protein 3D structure with a 3D structure viewer Molmil (19), and users can interactively examine genomic variants on protein structures (Figure 3C). The effects of SNVs on the RefSeq (20) protein sequences were determined according to the protein-coding regions on the GRCh37/hg19 reference genome, as described by the consensus CDS (CCDS) project (21). The atomic coordinates corresponding to amino acid changes were obtained based on the sequence alignments between the RefSeq proteins and PDB (22) proteins by using BLAST (23) at a threshold of sequence identity of 80% or more. We used DSSP (24) to calculate the secondary structure and accessible surface area (ASA) of the residues.

### Methylation/Transcriptome data

Summary statistics for the methylome and transcriptome were integrated into jMorp from iMETHYL, a genome browser developed by the Iwate Medical Megabank Organization (IMM) (25). These datasets include DNA methylation and gene expression information for three blood cell types (CD4^+^ T lymphocytes, monocytes, and neutrophils) and DNA methylation information for eight blood cell populations (peripheral blood mononuclear cells (PBMC), leukocytes, B lymphocytes, CD4^+^ T lymphocytes, CD8^+^ T lymphocytes, monocytes, natural killer cells, and neutrophils). Three blood cell types were obtained from approximately 100 participants in the TMM project through whole-genome bisulfite sequencing, RNA sequencing, and eight blood cell populations were obtained from 20 participants, respectively.

Users can refer to these summary statistics on a genome browser in jMorp. There are two categories of tracks for DNA methylation and gene expression—‘IMM Methylome & Transcriptome—three cell-types’ and ‘IMM Methylome & Transcriptome—eight cell-types’—corresponding to datasets of three cell types and eight cell populations, respectively. There are four tracks of DNA methylation information for each of the three cell types. On a track with the shortest name, such as ‘CpG_CD4T,’ clickable bars appear at the corresponding methylation sites. By clicking on the bars, the distribution of DNA methylation levels across 100 participants is displayed so that uses can refer to the interindividual variation of DNA methylation status. On the other tracks (e.g. CpG_CD4T_avg, CpG_CD4T_sd and CpG_CD4T_RI), the average or degree of interindividual variation in DNA methylation is represented by bar graphs. On these tracks, the landscape of DNA methylation is visualized on the genome browser and uses can find specific DNA methylation regions, such as the DNA methylation valley. RI represents the reference interval representing the interindividual variation of DNA methylation level, which is the difference between the 95th and 5th percentiles of the DNA methylation levels across individuals (26). For eight cell populations, only average DNA methylation levels are shown because a small sample size (n = 20) is insufficient to represent interindividual variation in DNA methylation. For gene expression information of each of the three cell types, a single track is available whereby the distribution, average, and standard deviation of gene expression levels (FPKM) across 100 participants are shown on a pop-up window. Some of the summary statistics mentioned above are downloadable and thus can be integrated into other independent datasets.

### Proteome data

Proteome data in jMorp 2020 is unchanged from the initial release in 2017 (14). Briefly, the proteome layer shows a list of 256 proteins, which were detected in plasma samples from 501 individuals by liquid chromatography followed by tandem mass-spectrometry, sorted by the detection rate in the samples. For each detected protein, the amino acid sequence, mass-to-charge ratio, charge and modifications of each detected peptide are shown in a separate page. Peptides including single amino acid changes due to non-synonymous genomic variants with minor allele frequency >5% were also detected and shown in the protein pages.

### Metabolome data

**Figure 4.**
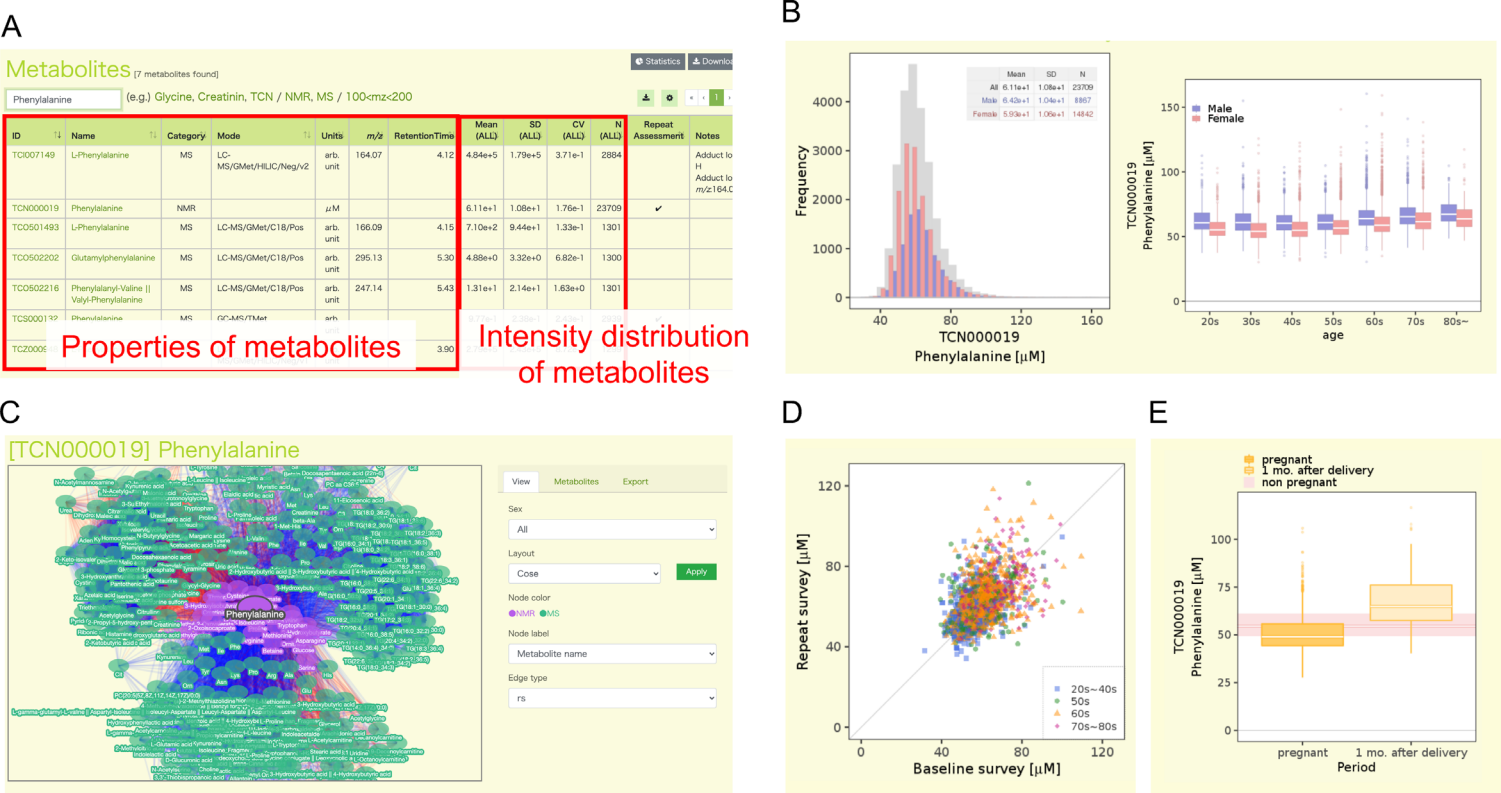
Overview of the metabolome section in jMorp. (**A**) Example of the search page of metabolites. The figure shows the metabolite table filtered by the word ‘Phenylalanine’. The left side of the table shows the basic properties of the metabolite, and the information about the concentration distribution of the metabolite is shown in the center. By clicking on the name of a metabolite in the table, users can move to the detailed page of the metabolite. (**B**) Distributions of the concentration of Phenylalanine (TCN000019) as an example. The plot on the left shows the distribution of the concentration of phenylalanine by gender, and the plot on the right shows that by age. (**C**) An example of the correlation network view. Nodes and edges on the network correspond to metabolite and correlation between two metabolites, respectively. The colors of the edges indicate the direction of the correlation (red: positive correlation, blue: negative correlation), and opacity of the edges indicate the strength of the correlation. (**D**) Change of Phenylalanine (TCN000019) concentration of same individual over time (3–4 years). One dot in the plot corresponds to one individual, and the horizontal and vertical axes indicate the concentration of phenylalanine in the first and second measurements, respectively. The dot color indicates the age of the individuals. (**E**) Difference of metabolite concentration between pregnant or after-birth periods. The two boxplots show the distribution of Phenylalanine (TCN000019) concentrations during pregnancy and one month after delivery, respectively. The distribution of phenylalanine concentration in non-pregnant individuals is shown in pink behind the two boxes.

The metabolome layer of jMorp mainly consists of two types of pages: table page with search function and a page for each metabolite. The table page includes compound IDs, compound names, platform categories (NMR or MS), units, MS modes (LC–MS, etc.), MS parameters (*m/z*, retention time), and basic statistics. Initially, entries sorted by compound ID are shown; this section is replaced with the search results after users carry out a search (Figure 4A). The search function is operated in the text box at the top of the table. Compound IDs and names in the search results are linked to each compound page. Notably, MS data can be searched for a range of *m/z* values. For example, a search with ‘100 < *m*/*z* < 200’ will show a compound list with *m/z* values ranging from 100 to 200. For MS data, in addition to *m/z* search, the absolute concentration (μmol/l) of each metabolite in the global quality control (gQC) plasma (pooled normal human plasma Na EDTA (Innovative Research, Parts No. IPLA-N, Lot 26393)) and/or reference plasma (NIST SRM 1950 (27)) is shown in the last column in the view of metabolites using the kit analyses, which are shown as TCBxxxxxx and TCMxxxxxx. When a user analyzes the gQC and/or NIST SRM 1950 plasma sample/s at the same time with their plasma samples by following the same protocol using the kit analysis, they can normalize the concentration and directly compare it to the value of metabolites in jMorp. All search results can be downloaded from the download icon at the right top corner of the table. In addition, basic statistical data (age, sex, and BMI) are included in the statistics summary page, which can be accessed from the ‘statistics’ icon at the right top corner of the table.

Each compound page includes icons for the following information: distributions in the population, age dependency of distribution, correlation between metabolites, difference of metabolite concentration between pregnant or after-birth periods, and change in metabolite concentration of the same individual over time (3–4 years). Each information can be accessed from each icon. In addition, links to other public compound databases (DB) are provided below. Links to HMDB (28), KEGG Compound (29) and LIPID MAPS (30,31) are shown if the focused compound is available in these databases.

The distributions of the compound concentration or abundance are shown for all (gray bars in Figure 4B), male (blue bars), and female (red bars) samples. To the left of the distributions, we show changes in metabolite abundance across age groups, where data are separated by sex. Age dependency of distribution is also shown by the box-plot for each age group. The correlation information can be represented by a table or network view. The correlation table includes a list of correlations between the focused metabolite and other metabolites among the population. In the table, Spearman's rank correlation coefficient (|*r*_s_| > 0.2) and p-value between two metabolites are shown. An interactive correlation network view is also available (Figure 4C). Correlation information is one of the main contents of jMorp. As far as we know, this is the first database of metabolite correlations among a healthy population. jMorp also provides information of metabolome data obtained from volunteers in follow-up survey and difference of metabolome during pregnancy. The TMM project is operating the follow-up survey (9), and those who have participated in the population‐based adult cohort once are asked to participate in the follow-up survey a few years later. We can observe changes in the concentrations of metabolites from the measurement results of those who participated in the two surveys. Figure 4D shows an example of age-related changes of the concentration of Phenylalanine (TCN000019). In the figure, a dot indicates an individual, and the horizontal and vertical axes indicate concentrations of a metabolite in first and second measurements, respectively. Figure 4E shows changes in the concentration of Phenylalanine (TCN000019) due to pregnancy. The Birth and Three-Generation Cohort Study in TMM project is a family-based cohort, in which pregnant women and their families participate. Metabolites of pregnant women were measured two times in pregnant period and one time at 1 month after delivery, and the two boxes in Figure 4E show the distribution of concentration during pregnancy and after delivery, respectively.

Finally, the five metabolite pages include the results of the metabolome genome-wide association study (MGWAS) results, shown by a Manhattan plot with variations in the compound across genotypes at the most significant variant. MGWAS information will be increased in future releases when we have obtained further MGWAS results.

### Repository of GWAS summary statistics data

Recently, meta-analysis has been a popular tool in genome-wide association studies (GWAS) (32–34). Meta-analysis integrates and analyzes the results of multiple GWAS analyses, thus allowing users to obtain more reliable results compared with single GWAS analysis. Many tools for meta-GWAS, for example, METAL (35) and GWAMA (36), have been developed. To facilitate meta-analysis, we implemented a repository of GWAS summary statistics files in jMorp. We collected GWAS analysis data within the TMM project and host summary statistics data in a uniform format similar to statistical files provided from the GWAS Catalog (37) database for reusability. The summary statistics files can be easily visualized on the jMorp website as Manhattan plots and QQ plots with the power of PheWeb (38) application. Currently, several GWAS statistics files, including MGWAS results described in Metabolome section above, are registered and linked to the corresponding entries in genome-omics layers in jMorp.

## METHODS

### Whole-genome analysis and construction of 8.3KJPN

#### Whole-genome sequencing

The 8.3KJPN was constructed based on whole-genome sequencing of 8,380 individuals in Japan (Supplementary Table S2). Genomic DNA extracted from the peripheral blood samples was used. Sequencing with HiSeq 2500 (Illumina) was performed as previously described (17). Sequencing with NovaSeq 6000 (Illumina) was carried out at TaKaRa Bio Co. Ltd using the following protocol: Library preparation was performed using TruSeq DNA PCR-Free Library Prep Kit with IDT for Illumina TruSeq DNA UD Indexes (Illumina) according to the manufacturer's 350 bp insert size standard protocol, and 150-bp paired-end sequencing was performed using NovaSeq 6000 S4 Reagent Kit with NovaSeq Xp 4-Lane Kit (Illumina).

We also used the DNBSEQ-G400 system (MGI Tech; Shenzhen, China) for whole-genome resequencing. The DNBSEQ-G400 system takes advantage of unique reaction chemistry: combinatorial probe-anchor synthesis (cPAS) corresponding to Illumina's sequence by synthesis (SBS) and DNA nanoball (DNB) formation corresponding to Illumina's bridge-PCR-based cluster generation. A single-stranded circular DNA library was prepared using MGIEasy PCR-Free DNA Library Prep Set Ver.1.0 and Ver.1.1 (MGI Tech) according to the manufacturer's 400 bp insert size standard protocol, followed by DNB formation based on the rolling circle amplification. Prepared DNB was loaded into the flow cell (DNBSEQ-G400RS Sequencing Flow Cell Ver.3.0), and cPAS-based 150-bp paired-end sequencing was performed with DNBSEQ-G400RS High-throughput Sequencing Set Ver.3.1 (MGI Tech).

#### Resequencing analysis of whole-genome sequence

The resequencing analysis for the sequences obtained by the sequencers was performed as described by Tadaka *et al.* (17) with minor modifications. In briefly, employed a workflow known as the GATK Best Practices workflow, which is becoming the standard procedure globally for whole-genome re-sequencing analysis. In the joint genotyping step, a total of 9,295 samples were simultaneously genotyped. We used KING (40) v2.2.5 software to perform pedigree estimation from 9,295 genotypes and extracted 8,380 unrelated samples. Finally, the 8.3KJPN frequency panel was constructed from the 8,380 unrelated samples.

Although we did not perform base quality score recalibration (BQSR) when we constructed 3.5KJPNv2, the predecessor of 8.3KJPN, we used BQSR as a step in the construction of 8.3KJPN. As described in our previous report (17), the 3.5KJPNv2 panel included only samples analyzed by the Illumina HiSeq 2500 system, and there was no significant difference in the results of joint genotyping with or without the BQSR process. However, we decided to apply BQSR for the construction of the 8.3KJPN panel because it contains samples analyzed by the Illumina HiSeq 2500 as well as by the Illumina NovaSeq 6000 system and MGI DNBSeq G400 system. The BQSR modifies the quality score of each base reported by the sequencer using known variant information, and this process is expected to be effective for reducing sequencer-specific bias (41).

### Methylation and transcriptome analyses

#### Sample information

DNA methylation and gene expression data for three blood cell types, including CD4^+^ T lymphocytes, monocytes, and neutrophils, were obtained from 102, 102, and 94 participants in the TMM project, respectively. DNA methylation and gene expression data for each cell type were obtained from identical participants. DNA methylation data for eight cell populations, including PBMC, leukocytes, B lymphocytes, CD4^+^ T lymphocytes, CD8^+^ T lymphocytes, monocytes, natural killer cells, and neutrophils, were obtained from 20 identical participants. Each cell type/population was isolated using an appropriate lysis buffer and antibodies (see http://imethyl.iwate-megabank.org for details). Genomic DNA was extracted from all samples, whereas RNA was extracted from 102 CD4^+^ T lymphocytes, 102 monocytes, and 94 neutrophils using the AllPrep DNA/RNA Micro Kit (Qiagen, Venlo, The Netherlands), following the manufacturer's instructions.

#### Procedures for DNA methylation analysis

Procedures for DNA methylation analyses were described by Hachiya *et al.* (26). Briefly, genomic DNA was subjected to bisulfite conversion and were sequenced on an Illumina HiSeq 2500 or HiSeq X. After adapter removal and a series of quality controls, sequence reads were mapped onto the GRCh37/hg19 human reference genome by NovoAlign v3.02.08 (Novocraft Technologies, Selangor Darul Ehsan, Malaysia). The DNA methylation level of each CpG site was calculated by dividing the number of unconverted cytosines by the total number of converted and unconverted cytosines aligned on the CpG site.

#### Procedures for gene expression analysis

The procedure for gene expression analyses was also described by Hachiya *et al.* (26). Using Superscript II reverse transcriptase (Thermo Fisher Scientific, Waltham, MA, USA), the total RNA was converted into cDNA, which was further applied for library preparation using the TruSeq RNA Sample Preparation Kit v2 (Illumina). The libraries were sequenced on an Illumina HiSeq 2500. Sequence reads were mapped onto the GRCh37/hg19 human reference genome using TopHat v2.0.13 (42), and FPKM values were calculated using cuffquant and cuffnorm programs in the Cufflinks package v2.2.1 (43).

### Metabolome analysis

#### NMR measurements

Details of sample preparation and NMR metabolome analysis were previously described elsewhere (12). In brief, plasma samples collected from cohort participants were stored at −80°C. Metabolites were extracted from 200 μl plasma samples using a standard methanol extraction procedure and were suspended in sodium phosphate buffer. All NMR experiments were performed at 298 K on a Bruker 600 MHz spectrometer (Bruker BioSpin, Germany). Standard 1D nuclear Overhauser effect spectroscopy (NOESY) and Carr-Purcell-Meiboom-Gill (CPMG) spectra were obtained for each sample. Data were analyzed using the Chenomx NMR Suite (Chenomx, Edmonton, Canada). Automatic quantification of each metabolite was performed using in-house software (Aoki *et al.* in preparation). The number of samples analyzed by NMR is shown in Supplementary Table S3.

#### MS measurements

The 2,270 cohort plasma samples for the wide targeted metabolomics (WT-Met) by gas chromatography triple quadrupole mass spectrometry (GC–MS/MS) analysis were prepared as described in previous studies (44–46). The sample was subjected to GC–MS/MS. The area ratio of each metabolite was calculated, and the intra-batch and inter-batch differences were corrected by the area ratio by the gQC analyses (47).

For the WT-Met by ultra-high performance liquid chromatography triple quadrupole mass spectrometry (UHPLC–MS/MS) analyses, 2,187 and 2,310 cohort plasma samples (10 μl, each) were prepared using the AbsoluteIDQ^®^ p180 kit and MxP^®^ Quant 500 kit (Biocrates Life Sciences AG, Innsbruck, Austria), respectively (48,49). The optimal conditions of UHPLC–MS/MS for the LC mode and FIA mode and the ionization parameters, ion transfer voltages/temperatures, and the detection of *m/z* pairs of precursor and product ions in multiple reaction monitoring mode by MS were automatically set based on the instructions for each kit. The quantified values (μmol/l) were calculated and normalized according to the manufacturer's protocol using MetIDQ Oxygen software (Biocrates). The number of samples analyzed by MS is also shown in Supplementary Table S3.

## DATA AVAILABILITY

The jMorp web database is freely available from https://jmorp.megabank.tohoku.ac.jp. The information stored in the database will only be provided to persons who have agreed to jMorp's Conditions of Use (https://jmorp.megabank.tohoku.ac.jp/help/conditions-of-use).

## Supplementary Material

gkaa1034_Supplemental_File
