# Supplementary material for: jMorp updates in 2020: large enhancement of multi-omics data resources on the general Japanese population
Source: Nucleic Acids Res. 2020 Nov 12;49(D1):D536–44. doi: 10.1093/nar/gkaa1034 (PMC7779038; doi:10.1093/nar/gkaa1034)
Supplement: gkaa1034_Supplemental_File [file gkaa1034_supplemental_file.pdf]

jMorp updates in 2020: Large enhancement of multi-omics data resources of a general Japanese population (Supplementary Materials)

Shu Tadaka[1], Eiji Hishinuma[1,2], Shohei Komaki[3], Ikuko N. Motoike[1,5], Junko Kawashima[1], Daisuke Saigusa[1,4], Jin Inoue[1], Jun Takayama[1,2], Yasunobu Okamura[1,2], Yuichi Aoki[1,5], Matsuyuki Shirota[1,2,4,5], Akihito Otsuki[1,4], Fumiki Katsuoka[1,4], Atsushi Shimizu[3,6], Gen Tamiya[1,4], Seizo Koshiba[1,2], Makoto Sasaki[3,6], Masayuki Yamamoto[1,2,4], Kengo Kinoshita[1,2,5,*]

1. Tohoku Medical Megabank Organization, Tohoku University, 2-1 Seiryo-machi, Aoba-ku, Sendai, Miyagi 980-8573, Japan
2. Tohoku University Advanced Research Center for Innovations in Next-Generation Medicine, Tohoku University, 2-1 Seiryo-machi, Aoba-ku, Sendai, Miyagi 980-8573, Japan
3. Iwate Tohoku Medical Megabank Organization, Disaster Reconstruction Center, Iwate Medical University, 1-1-1 Idaidori, Yahaba-cho, Shiwa-gun, Iwate 028-3694, Japan
4. Graduate School of Medicine, Tohoku University, 2-1 Seiryo-machi, Aoba-ku, Sendai, Miyagi 980-8575, Japan
5. Graduate School of Information Sciences, Tohoku University, 6-3-09 Aramaki aza Aoba, Aoba-ku, Sendai, Miyagi 980-8579, Japan
6. Institute for Biomedical Sciences, Iwate Medical University, 1-1-1 Idaidori, Yahaba-cho, Shiwa-gun, Iwate 028-3694, Japan

* To whom correspondence should be addressed.

Supplementary Table 1: basic statistics of the data in jMorp

(a) Summary of main datasets in jMorp and number of samples per dataset. The numbers in the metabolome, proteome, transcriptome, methylome, and genome variation rows represent the number of specimens analyzed to construct each panel. The number written in the Genome Sequence row is the number of specimens used in the *de novo* assembly to construct the reference sequence.

**Data Types**	**Description**	**# of samples analyzed and included in each dataset by year**					
		**2015**	**2016**	**2017**	**2016**	**2019**	**2020**
**Metabolome**	Information on plasma metabolome in up to 13,000	501	1,008	5,093	10,719	13,729	23,709
**Proteome**	Information on the plasma proteome of approximately 500 Japanese	501	501	501	501	501	501
**Transcriptome** / **Methylome**	WGBS, WGS and RNA-Seq analysis of CD4T cells and monocytes isolated from approximately 100 Japanese					298	298
**Genome Variation**	Information on genome variant frequencies from the WGS analysis of approximately 8,300 Japanese				3,552	4,773	8,380
**Genome Sequence**	Japanese reference sequence obtained by *de-novo* assembly of the three Japanese men's sequences				3	3	3

(b) Summary of supplementary datasets in jMorp.

**Data Types**	**Description**
**Genetic Map**	Linkage disequilibrium maps calculated from 192 haploid genomes
**Japonica Array Marker list**	Marker information on the SNP array "Japonica Array" constructed based on the TMM’s Whole Genome Reference Panel 3.5KJPNv2
**GWAS Summary Statistics**	A repository of statistics data of GWAS analyses performed using TMM's data

Supplementary Table 2: Number of samples included in 8.3KJPN frequency panel by cohort and analysis platform

Participant Group	Sequencer	# of participants
TMM Project (participants in Miyagi and Iwate prefectures)	Illumina HiSeq 2500	3,619
	Illumina HiSeq X Five	71
	Illumina NovaSeq 6000	3,691
	MGI DNBSeq G400	577
Nagahama Study	Illumina HiSeq 2500	65
J-MICC Study	Illumina HiSeq 2500	60
Individuals recruited in National Cancer Center Hospital, Japan	Illumina HiSeq 2500	47
Individuals recruited in Nagasaki Medical Cencer, Japan	Illumina HiSeq 2500	191
Individuals recruited in department of Ophthalmology, Osaka University, Japan	Illumina HiSeq 2500	30
Individuals recruited in Osaka University Center for Twin Research, Japan	Illumina HiSeq 2500	29
**Total**		**8,380**

Supplementary Table 3: Number of samples included in the metabolome dataset by data type and the analysis method

Data Type	Category	Global or Targeted?	Mode	# of samples	# of metabolites
Results of metabolome analysis for the general population	NMR	Targeted	-	23,709	45
	LC-MS	Global	HILIC-neg v2	2,998	165
	LC-MS	Global	HILIC-neg v1	1,301	269
	LC-MS	Targeted	-	2,411	4
	LC-MS	Targeted	Kit180	1,493	110
	LC-MS	Targeted	Kit500	2,374	421
	GC-MS	Targeted	-	2,932	169
Time series variation	NMR	Targeted	-	1,685	44
	LC-MS	Targeted	Kit180	581	110
	GC-MS	Targeted	-	639	165
Changes due to pregnancy	NMR	Targeted	-	2,074	45
